# Inhibitors of Na^+^/K^+^ ATPase exhibit antitumor effects on multicellular tumor spheroids of hepatocellular carcinoma

**DOI:** 10.1038/s41598-020-62134-4

**Published:** 2020-03-24

**Authors:** Yeonhwa Song, Su-Yeon Lee, Sanghwa Kim, Inhee Choi, Se-Hyuk Kim, David Shum, Jinyeong Heo, A-Ram Kim, Kang Mo Kim, Haeng Ran Seo

**Affiliations:** 1 Cancer Biology Laboratory, Institut Pasteur Korea, 16, Daewangpangyo-ro 712 beon-gil, Bundang-gu, Seongnam-si, Gyeonggi-do 13488 Korea; 2 Medicinal Chemistry, Institut Pasteur Korea, 16, Daewangpangyo-ro 712 beon-gil, Bundang-gu, Seongnam-si, Gyeonggi-do 13488 Korea; 3 Screening Discovery Platform, Institut Pasteur Korea, 16, Daewangpangyo-ro 712 beon-gil, Bundang-gu, Seongnam-si, Gyeonggi-do 13488 Korea; 4 Department of Gastroenterology, Asan Liver Center, Asan Medical Center, University of Ulsan College of Medicine, Olympic-ro 43-gil 88, Songpa-gu, Seoul 05505 Korea

**Keywords:** Cancer microenvironment, Chemotherapy, Tumour heterogeneity, High-throughput screening, Phenotypic screening

## Abstract

Hepatocellular carcinoma (HCC), one of the most common malignant cancers worldwide, is associated with substantial mortality. Because HCCs have strong resistance to conventional chemotherapeutic agents, novel therapeutic strategies are needed to improve survival in patients with HCC. The multicellular tumor spheroid (MCTS) model is a powerful method for anticancer research because of its ability to mimic the complexity and heterogeneity of tumor tissue, the three-dimensional cellular context of tumor tissue, and the pathophysiological gradients of *in vivo* tumors. However, it is difficult to obtain meaningful results from the MCTS model without considering the conditions of clinical tumors. We, therefore, provided a proof of concept to determine whether spheroid models simulate *in vivo* tumor microenvironments. Through a high-throughput screening for HCC therapy using the MCTS model, we selected inhibitors of Na^+^/K^+^-ATPase (ouabain and digoxin) that could suppress cell growth and migration via inhibition of the epithelial-mesenchymal transition of HCC *in vivo* and *in vitro*. The results showed that this model provides a new paradigm for high-throughput drug screening and will significantly improve the efficiency of identifying new drugs for HCC treatment. Through utilization of MCTS models, here we found that inhibitors of Na^+^/K^+^-ATPase may be feasible as a novel target to sensitize HCC cells.

## Introduction

Cancers are still the leading cause of human death, and hepatocellular carcinoma (HCC) is one of the most serious of these cancers^1^. Typical first-line therapy for HCC involves surgical resection of the tumor, and second-line therapy involves several treatment with sorafenib, a multi-tyrosine protein kinase inhibitor for metastatic or unresectable HCC^2^. However, both therapies are often not effective for treating the patient. The major causes of HCC are alcoholism and chronic infection by hepatitis B or C, as well as metastasis from tumors elsewhere in the body. The cumulative 3-year recurrence after resection is approximately 80%, usually resulting in a high mortality rate^3^. From a clinical standpoint, awareness of the clinical manifestations of the patient during therapy is very important. Researchers have tried to identify target genes and drug candidates for HCC diagnosis and treatment; however, the development of targeted drugs has not yet significantly improved the prognosis^4^.

Because of the difficulties in correlating *in vitro* with *in vivo* conditions of cancer, we developed a new model to screen drugs in a biologically relevant context. The tumor microenvironment (TME) has important physiological roles in cellular differentiation and tumorigenesis, as well as metastasis and therapeutic efficacy^5–7^. It is difficult to obtain relevant results about the formation of the TME without considering clinical tumor conditions^8^. Presently, two-dimensional (2D) cell–based assay models have dominated preclinical cancer drug discovery efforts. However, 2D cell–based models fail to predict *in vivo* efficacy, contributing to a lower success percentage in translation of the new drug for clinical use. Hence, we thought that a 2D assay system would not be beneficial because the resulting data could not be utilized for translational research. In contrast, a complex three-dimensional (3D) cell culture system better replicates the 3D cellular context and simulates therapeutically relevant parameters of *in vivo* tumors, such as pH and oxygen gradients, the penetration of growth factors, and the distribution of proliferating/necrotic cells^9–11^. In particular, liver cells in a 3D culture system, compared with a 2D culture system, better perform numerous liver functions, including albumin and urea synthesis, bile secretion, and cell polarization^12,13^. The benefit of testing drugs in a 3D cell culture system is that cells form multiple layers rather than a monolayer found in a 2D system. When testing a drug in a 2D culture system, the drug needs only to diffuse a short distance across the cell membrane to reach its target. A 3D system better replicates an *in vivo* tumor because the drug must diffuse across multiple layers of cells to reach its target. Based on these considerations, we developed a 3D TME model to screen possible drugs for HCC.

Recently, the multicellular tumor spheroid (MCTS) model has emerged as a powerful method to mimic the *in vivo* properties of a tumor, replicate tumor complexity, and predict drug efficacies for anticancer research.

In our previous results, we reported the reciprocal action between tumor and stromal cells (i.e., fibroblasts, vascular endothelial cells, hepatic stellate cells, and immune cells) in a spheroid model system, which reproduced important tumor parameters such as sensitivity to chemotherapy, migration, and proliferation^14,15^. Crosstalk between tumor and stromal cells could alter the expression of extracellular matrix molecules and epithelial-mesenchymal transition (EMT)–related proteins in the MCTS model^16,17^. Hence, the MCTS model is an appropriate system that mimics the behavior of the EMT and the propagation of cancer cells *in vivo*.

In the present study, we improved the culture conditions and provided proof of concept to determine whether the MCTS model closely replicates the TME *in vivo*. To apply the MCTS model to a high-throughput screening system, we first developed the most efficient method for producing 3D spheroids, because the formation of 3D spheroids is an expensive, cumbersome, and complicated process. Despite these difficulties, we established and validated a highly reproducible MCTS-based high-throughput screening system with homogenously sized and configured single spheroids in multiwell plates to successfully screen possible drugs for HCC therapy. This screening system provides a new model for anticancer drug screening that significantly improves the efficiency and reliability of identifying new drugs for HCC treatment.

## Materials and Methods

### Cell lines and culture conditions

Seven human HCC cell lines—Huh7, Hep3B, HepG2, PLC/PRF/5, SNU449, SNU475, and Huh6—were obtained from the Korean Cell Line Bank (Seoul, Korea), and the human immortalized hepatocyte Fa2N-4 cell line was purchased from Xenotech (Lenexa, KS, USA). The Huh7.5-red fluorescent protein-(RFP)-NLS-IPS reporter cell line (Huh7.5) was kindly provided by Dr. Marc Windisch (Institut Pasteur Korea, Gyeonggi-do, Korea). Of the stromal cell lines, WI38 human fibroblasts were purchased from ATCC (Manassas, VA, USA); LX2 human hepatic stellate cells (HSCs), Merck Millipore (Darmstadt, Germany); and human umbilical vein endothelial cells (HUVECs), PromoCells (Heidelberg, Germany). All cells were maintained at 37 °C in a humidified atmosphere of 5% CO_2_.

HCC cell lines were cultured in Dulbecco’s modified Eagle’s medium (DMEM; Welgene, Daegu, Korea) supplemented with heat-inactivated 10% fetal bovine serum (FBS; Gibco, Grand Island, NY, USA) and 1 × penicillin-streptomycin (P/S; Gibco). WI38 cells were cultured in minimum essential media (Welgene) supplemented with heat-inactivated 10% FBS and 1× P/S. LX2 cells were cultured in DMEM supplemented with heat-inactivated 2% FBS and 1× P/S. For HUVEC culturing, endothelial basal medium was purchased from PromoCells. Fa2N-4 cells were plated in collagen-coated plates (BD Biosciences, San Jose, CA, USA) in serum-containing plating medium (XenoTech), and after cell attachment (approximately 3–6 hours), serum-containing plating medium was replaced with supporting culture medium (XenoTech).

### Primary culture of HCC cells

Immediately after surgery, a portion of the tumor was immersed in Hank’s balanced salt solution (HBSS; Gibco) and transported from the operating room at 0 °C to the laboratory. The specimens were collected under sterile conditions and rinsed 2–3 times with HBSS free of calcium and magnesium to remove the blood. Next, the liver samples were excised, cut into small fragments, gently dispersed, and placed in HBSS containing 0.03% Pronase (Gibco), 0.05% type IV collagenase (Gibco), and 0.01% deoxyribonuclease (DNase bovine pancreas; Gibco) for 20 minutes at 37 °C. The resulting suspension was filtered through a 100-μm nylon filter (BD Falcon, Franklin Lakes, NJ, USA) and centrifuged at 50 × g for 2 minutes at 4 °C to obtain hepatocytes. The pellet was washed twice in HBSS containing 0.005% DNase. The final cell suspensions were cultured in collagen-coated T25 flasks (BD Falcon) in F12/DMEM (Gibco), supplemented with 20% FBS, 1% non-essential amino acid (Gibco), 1% glutamine, and 1% P/S at 37 °C in a humidified atmosphere of 5% CO_2_. The medium was changed 24 hours after seeding to remove dead cells and debris. When confluency reached 70–80%, the cells were replated using a 1:1 mixture of DMEM and F12/DMEM with supplements. After five passages, the cells were grown in DMEM supplemented with heat-inactivated 10% FBS and 1% P/S. Confluent cells were trypsinized, counted, and split 1:3–1:5 at every passage. After the cell lines were maintained over 30 passages, they were collected and stored in liquid nitrogen.

### Ethics approval and patient consent

This study was conducted in accordance with the Declaration of Helsinki. It was approved by the Human Research Ethics Committee of the Asan Medical Center. The institutional review board at the Asan Medical Center complied with related laws such as the International Council for Harmonization of Technical Requirements for Pharmaceuticals for Human Use (ICH), Korea Food and Drug Administration’s Good Clinical Practice guidelines (KGCP), and bioethics and safety acts. Written informed consent for the use of the tissues for research was obtained from patients at the time of procurement of tumor specimens.

### Immunofluorescence

Liver cancer samples from formalin-fixed and paraffin-embedded tissues were analyzed on an AccuMax Array system (Petagen, Seoul, Korea). For immunofluorescence, the arrayed slides were used for antibody immunostaining of anti-human CD31 (ab28364, 1:500; Abcam, Cambridge, UK), anti-human CD44 (ab51037, 1:500, Abcam, Cambridge, UK), anti-human fibroblast activation protein (FAP, ab28244, 1:500; Abcam), and anti-Ki67 (ab15580, 1:500; Abcam). Deparaffinization and rehydration were performed using xylene and ethanol (Sigma-Aldrich, St Louis, MO, USA), and the pretreated slides were incubated in 3% H_2_O_2_ (Sigma-Aldrich) for 13 minutes to remove endogenous peroxidase activity. The tissues were incubated with antibodies for 16 hours at 4 °C, washed with Dulbecco’s phosphate-buffered saline (DPBS; Welgene) for 10 minutes, and then incubated for 2 hours at room temperature with goat anti-rabbit IgG-conjugated Alexa Fluor 633 (Invitrogen, Eugene, OR, USA) and goat anti-mouse IgG-conjugated Alexa Fluor 488 (Invitrogen) secondary antibodies. After three washes with DPBS, the coverslips were mounted onto microscope slides using VECTASHIELD mounting medium with 4'6-diamidino-2-phenylindole (Vector Laboratories, Burlingame, CA, USA). The slides were analyzed using the Operetta High-Content Screening System (Perkin Elmer, Waltham, MA, USA).

### Generation of tumor spheroids

To generate tumor spheroids, cells suspended in complete media were seeded at a density of 6 × 10^3^ cells/well, depending on the proportions of cancer and stromal cells in 384- or 96-well round bottom ultra-low attachment microplates (Corning Life Sciences, Amsterdam, Netherlands). The plates were incubated for 3 days at 37 °C in a humidified atmosphere of 5% CO_2_. After 3 days, 10 µM of cytotoxic or anticancer drugs were added and incubated for an additional 7 days.

### Spheroid migration assay

Before generating the spheroids, LX2 cells, WI38 cells, and HUVECs were stained with Vybrant DiD (633 nm), DiI (546 nm), or Dio (488 nm) solution (Invitrogen) at a 1:500 ratio in complete media for 20 minutes in a 37 °C incubator. After washing with DPBS, the spheroids were formed over 3 days, and each spheroid was transferred to a collagen I-coated 384-well plate (Greiner Bio-one, Monroe, NC, USA) and cultured for 4 days in complete media. The medium was changed once at 2 days after transferring. After 4 days, the cells were fixed in 4% paraformaldehyde for 30 minutes at room temperature and washed twice with DPBS. For nuclei staining, the cells were incubated with Hoechst 33342 (Invitrogen) at a 1:500 ratio in DBPS for 10 minutes at room temperature. After washing twice with DPBS, fluorescence images were captured according to the optimal excitation and emission wavelengths of each probe. To capture the entire images, 25 image fields (starting at the center of the well) were collected from each well using a high-content screening system with a 10× objective.

### Xenograft study

To generate MCTSs, Huh7.5 cells were seeded with stromal cells (LX2 cells, WI38 cells, or HUVECs) at a density of 1 × 10^4^ cells/well in 96-well round bottom ultra-low attachment microplates. The plates were incubated for 3 days at 37 °C in a humidified atmosphere of 5% CO_2_. A total of 1 × 10^6^ cells from 2D or 3D culture systems with 0.1 mL of Matrigel (matrix growth factor reduced; BD Biosciences, San Jose, CA, USA) were injected subcutaneously in 5-week old male BALB/c nude mice (Central Lab. Animal Inc., Seoul, Korea). The xenograft mice were randomly classified for four groups (4–5 mice per group) and treated the compounds including sorafenib (10mpk), ouabain and digoxin (2mpk each group) in this study. The treatment of sorafenib was injected by intraperitoneal (i.p.) for twice a week and the treatment of ouabain and digoxin were injected by oral administration for everyday during experimental periods. The animals were weighed and the tumor sizes were measured with calipers twice a week. The tumor volume (mm^3^) was calculated using the following formula: volume = (width)^2^ × length/2. There were seven mice studied in each group. The animal protocol was approved by the institutional animal care and use committee of ASAN medical center and conducted strictly in accordance to the national institute of health guide for the care and use of laboratory animals.

### Histological analysis and immunohistochemistry

Tumor tissues were fixed in 4% paraformaldehyde, cut into 4-μm paraffin-embedded sections, and stained with hematoxylin and eosin (H&E) and Masson’s trichrome. For immunohistochemistry, after deparaffinization and dehydration, antigen retrieval was performed by boiling the sections in 10 mM citric acid buffer (pH 6.0) for 15 minutes. Antibodies used for immunohistochemistry included those against anti-human α-SMA (ab5694, 1:1,000; Abcam) and epidermal growth factor receptor (EGFR, 3777 S, 1:1,000; Cell signaling). The results were viewed with a microscope (Nikon, Tokyo, Japan).

### Biological and chemical informatics analysis

Based on the microarray data, functional enrichment analyses of genes with >2-fold change (absolute) were performed using Funrich software. We assessed which genes were significantly enriched or depleted in molecular functions, biological processes, and biological pathways from the microarray analyses of the lysates from the Huh7.5 spheroids and the MCTSs. Differentially expressed genes were functionally categorized to explain overrepresented molecular functions, biological processes, and biological pathways^18^. The information on pharmacological actions of each compound was combined from the ChemIDPlus, MESH, and SciFinder databases, as well as information provided from the vendors. All pharmacological actions were accounted for and were not limited to the major actions. Once relevant information was collected, pharmacological actions were manually reassessed to categorize all compounds into 69 different pharmacological actions.

### High-throughput screening

A library of 4,763 compounds was assembled from Tocris Bioscience (Avonmouth, Bristol, UK), Selleck Chemicals (Houston, TX, USA), LOPAC (St Louis, MO, USA), and Prestwick Chemical (Washington, DC, USA) and screened in the HCC- MCTS model at a final concentration of 10 μM in 0.5% DMSO (v/v). To produce spheroids, four cell lines (Huh7.5 cells, LX2 cells, WI38 cells, and HUVECs) were seeded at a density of 6 × 10^3^ cells/well into 96-well round bottom ultra-low attachment microplates. To facilitate cell aggregation, the plates with the cell suspensions were centrifuged at 1,000 rpm for 1 minute before incubation. The plates were then incubated at 37 °C in a humidified atmosphere of 5% CO_2_ for 3 days. For the testing of compounds, a 2-µL sample of each compound was transferred into an intermediate 384-well polypropylene plate (Greiner Bio-one) using a liquid handler (Apricot Personal Pipettor; Apricot Design, Covina, CA, USA). The compounds were mixed with 78 µL complete medium per well. Subsequently, a 20-µL sample of each compound was dispensed into each well of a 96-well assay plate. The plates were then incubated at 37 °C in a humidified atmosphere of 5% CO_2_ for 7 days. Controls were added to each assay plate with sorafenib at its IC_50_ concentration as a low control and 0.5% DMSO (v/v) as a high control. After 7 days, spheroid images were acquired using a high-content screening system. The size and intensity of RFP signals in spheroids were measured using a self-developed algorithm. Hit compounds were selected using a threshold based on 3σ (standard deviation) from the IC_50_ of sorafenib.

### Statistical analysis

All experiments were performed at least three times. The results are expressed as the mean ± standard deviation (SD). Statistical analyses were performed using Student’s *t*-test.

## Results

### MCTS is a model system that mimics the tumor microenvironment

In our previous study, we produced simple MCTS models with HCC cell lines and different types of stromal cells including LX2 HSCs, WI38 human fibroblasts, and HUVECs to model tumor complexity and heterogeneity *in vitro*. The interactions between each type of stromal cells and the HCC cells facilitated the compactness of the tumor spheroids and increased chemoresistance and HCC cell migration^15^. However, these previous MCTS models were not derived from a mixture of HCC cells with all HSCs, fibroblasts, and vascular endothelial cells.

**Figure 1:**
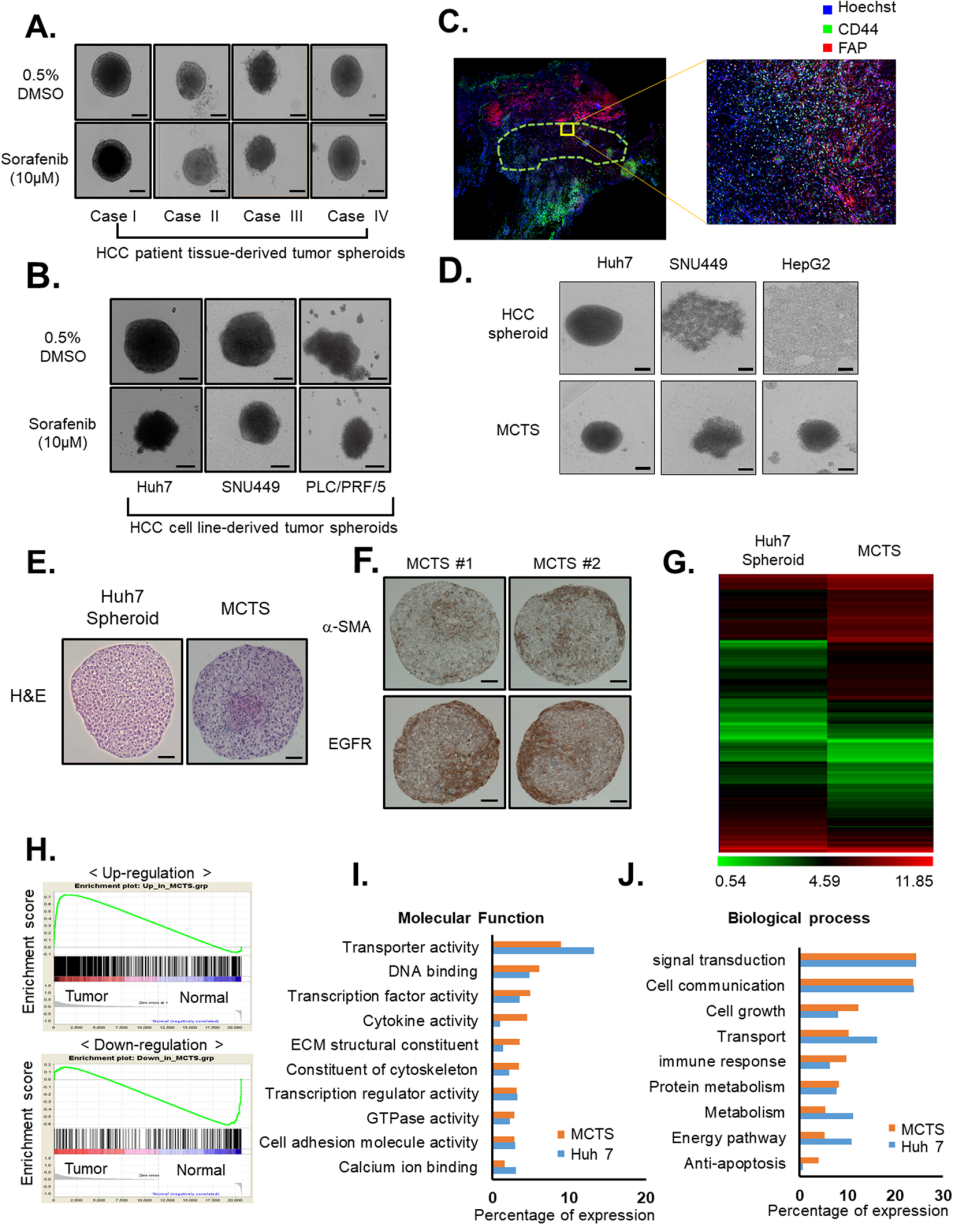
Establishment of a multicellular tumor spheroid (MCTS) model mimicking the microenvironment of hepatocellular carcinoma (HCC) patient tissues. (**A**,**B**) Drug sensitivities to 10 µM sorafenib in tumor spheroids using HCC patient-derived tumor spheroids (**A**) and HCC cell lines (Huh7, SNU449, and PLC/PRF/5) (**B**). (**C**) Representative histochemical images of CD44 (green) and FAP (red) expression after Hoechst 33342 staining for nuclei in tissues derived from patients with HCC. (**D**) Morphology of spheroids using HCC cell lines (Huh7, SNU449, and HepG2) with (MCTS) or without stromal cells (HCC spheroids). (**E**) Hematoxylin & eosin staining of Huh7 spheroid and MCTS. (**F**) Immunohistochemical analysis of epidermal growth factor receptor (EGFR) and α-SMA of consecutive sections of the MCTS model generated from HCC cells co-cultured with human stromal cells (hepatic stellate cells, fibroblasts, and vascular endothelial cells). (**G**) A gene expression heat map representing the fold-change ratios of the MCTS model versus tumor spheroids. (**H**) A gene-set enrichment analysis plot of upregulated genes (upper panel) or down-regulated genes (lower panel) of the MCTS model versus patients with liver cancer (GSE64041). (**I**,**J**) Horizontal bar graphs of molecular functions (**I**) and biological processes (**J**) overrepresented in highly enriched genes of the Huh7 spheroids (blue) and MCTS (orange) lysates. All images were obtained using the Operetta High-Content Screening (HCS) system and bright-field microscope. Data are shown as the means and standard deviation (SD) of triplicate experiments. Scale bar: 200 µm.

In the present study, we aimed to develop more complicated MCTS models to recapitulate the *in vivo* TME of HCC. Before the development of the MCTS models, we performed a comparison study of drug sensitivities between tumor spheroids and patient-derived HCC tumor spheroids after treatment with 10 µM sorafenib. The size of patient-derived tumor spheroids was not changed by sorafenib treatment [Fig. 1A]. However, the size of HCC cell line-derived spheroids was significantly reduced by treatment with sorafenib, relative to patient-derived tumor spheroids [Fig. 1B]. We analyzed the composition of tissues from patients with liver cancer using immunofluorescence probes for FAP (a marker for fibrosis) and CD44 (a marker for cancer cells). The results showed that tissues from patients with liver cancer were composed of certain percentages of stromal cells that can cause fibrosis of tissue such as HSCs, fibroblasts, vascular endothelial cells, and HCC cells [Fig. 1C, Supplementary Fig. 1]. These results suggested the possibility that crosstalk between stromal cells that can cause fibrosis of tissue and that HCC cells induce chemoresistance in HCC patient tissue-derived tumor spheroids.

There are four basic cell types in the liver: hepatocytes, which are parenchymal cells; and human HSCs, Kupffer cells, and endothelial cells, all of which are non-parenchymal cells. In the present study, we established more complicated MCTS models derived from a mixture of HCC cells and stromal cells that can cause fibrosis representatively HSCs, fibroblasts, and vascular endothelial cells to replicate the *in vivo* TME of liver cancer, because liver cancer involves extensive fibrosis.

To generate model tumor complexity and heterogeneity *in vitro*, HCC cell lines (Huh7, SNU449 and HepG2cells) were grown together with WI38 cells human fibroblasts, LX2 cell human HSCs, and HUVECs in MCTS models. Intriguingly, co-culture with HCCs and stromal cells in MCTS exhibited significantly induced enhancement of spheroid compactness and rigidness [Fig. 1D].

The MCTS model is a powerful tool for anticancer research because it mimics the complexity and heterogeneity of tumor tissues and contains a 3D cellular structure and the pathophysiological gradients of *in vivo* tumors. However, it is difficult to obtain valuable results if the MCTS models are used without considering clinical tumor conditions. We therefore determined if our models closely replicated *in vivo* TMEs.

Although a 3D system with cancer cell lines cannot recapitulate the genetic heterogeneity found in tumors, modeling cellular heterogeneity can be achieved to a certain degree with heterotypic multicellular models. The internal structures between the MCTS model and tumor spheroids alone were compared through H&E staining. Both spheroids displayed different patterns of H&E staining in the spheroid core [Fig. 1E]. The results of the immunohistological staining for α-SMA indicated an even more mesenchymal phenotype of fibroblasts, reflecting a mesenchymal-to-mesenchymal transition via the EMT and endothelial-mesenchymal transition (EndMT) of stromal cells in the TME. Expression of α-SMA was compartmentalized and primarily distributed at the center of the MCTS. 3D co-culture between HCC and stromal cells lead to an upregulation of the epidermal growth factor receptor (EGFR) on the surface of the MCTS [Fig. 1F].

Then, gene expression profiling was performed on the MCTS model systems to normalize to the profiles of tumor spheroids. We identified 1,596 differentially expressed genes between the MCTS and tumor spheroids, with a cutoff of >2-fold difference [Fig. 1G]. To demonstrate a MCTS model-specific signature that implicated it as the phenotype of patient-derived HCC tissues, we performed gene-set enrichment analyses (GSEA) using the Gene Expression Omnibus datasets from 60 HCC biopsies from an unselected patient population with all tumor stages (GSE64041). The results showed that gene expression profiling of the MCTS was more highly enriched in HCC tumor tissues than that of tumor spheroids, suggesting that the MCTS model is an effective tool for mimicking *in vivo* HCC microenvironments [Fig. 1H].

According to functional enrichment analyses that compared the number of molecular functions between Huh7.5 spheroids and MCTS models, genes that were involved in constituent extracellular matrix structural functions, structural constituents of the cytoskeleton, cytokine activity, transcription factor activity, and DNA binding were more highly overexpressed in the MCTS model than in the Huh7 spheroids [Fig. 1I]. In biological processes, genes related to the regulation of nucleobases, nucleoside, nucleotides, nucleic acid metabolism, cell growth, immune response, and anti-apoptotic responses were more enriched in the MCTS model [Fig. 1J]. Furthermore, genes related to the integrin family that were involved in cell surface interactions were more enriched in the MCTS model [Supplementary Fig. 2; Table 1].

**Table 1:** Summary of markedly enriched Molecular function, Biological processes, and Biological Pathway from the microarray analysis of MCTS model and Huh7 cells.

Functions	Enriched in the MCTS model	Enriched in the Huh7 cells
Molecular Function	Structural constituent of cytoskeleton	Calcium ion binding
	Extracellular matrix structural constituent	Transporter activity
	Cytokine activity	
	Transcription factor activity	
	DNA binding	
Biological Processes	Anti-apoptosis	Energy pathways
	Immune response	Metabolism
	Cell growth and/or maintenance	Transport
	Regulation of nucleobase, nucleoside, nucleotide and nucleic acid metabolism	
Biological Pathway	Integrin family cell surface interactions	

Biological processes and pathways in fold quantity-based analyses are more detailed. In the MCTS model, genes that were involved in cofactor binding, cytokine activity, nucleic acid binding, and extracellular matrix structural constituents were enriched. Notably, genes involved in glucosidase activity were greatly depleted (−15-fold changes), which should be further investigated [Supplementary Fig. 3]. Regarding biological processes, genes that were involved in peptide metabolism, regulation of endocytosis, anti-apoptosis, muscle contraction, cell-cell adhesion, and certain signaling pathways were enriched >2-fold [Supplementary Fig. 4]. Biological pathways involved in liver pathology and functions, such as Hedgehog signaling events mediated by Gli proteins, synthesis, secretion, and deacylation of ghrelin, and Vitamin A uptake in enterocytes, were depleted in the MCTS models [Supplementary Fig. 5].

### Reciprocal crosstalk between HCC and stromal cells promotes sorafenib resistance and HCC migration in the MCTS model

In a previous study, we suggested patient-derived MCTSs as the best methodology to screen potential cancer drugs for patients with HCC through comparison studies of chemosensitivity using three culture systems: monolayer culture system, tumor spheroids, and MCTSs of HCC patient-derived cells^19^.

**Figure 2:**
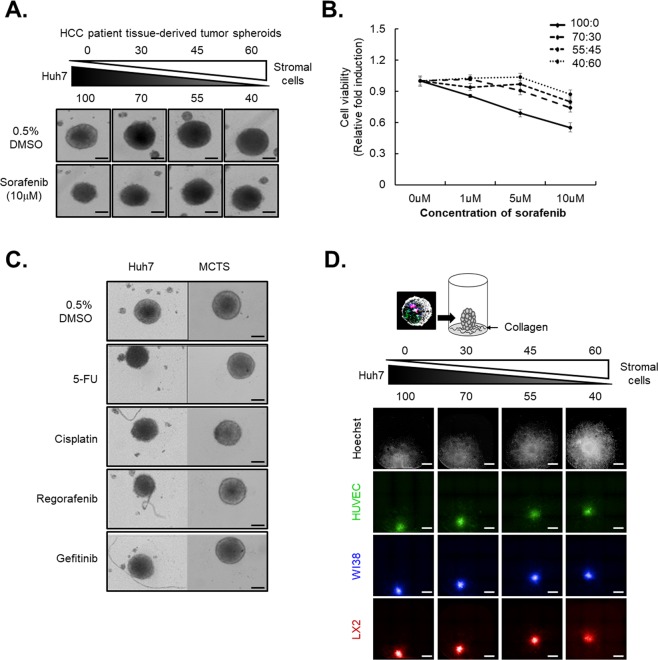
Reciprocal crosstalk between hepatocellular carcinoma (HCC) and stromal cells and drug resistance in multicellular tumor spheroid (MCTS). (**A**) Drug sensitivities to 10 µM sorafenib in different compositional MCTS models with different proportions of HCC and stromal cells (hepatic stellate cells, fibroblasts, and vascular endothelial cells). (**B**) Different cell viabilities after treatments with the indicated concentrations of sorafenib in MCTS models with different compositions of HCC and stromal cells using the resazurin assay. (**C**) Drug sensitivities to anticancer drugs (5-FU, cisplatin, regorafenib, gefitinib) between spheroid and MCTS models. (**D**) HCC cell migration in MCTS models with different compositions of HCC and stromal cells (green for HUVEC, blue for WI38, and red for LX2) after reattachment onto the collagen-coated plates in the complete media. Scale bar: 200 µm.

Based on the previous study, we generated different MCTS models with different proportions of HCC cells and stromal cells (i.e., HSCs, fibroblasts, and vascular endothelial cells) to determine the possible effects of crosstalk between stromal cells and HCCs on chemoresistance and cell migration. We also selected the most appropriate MCTS models for HCC drug screening by characterizing the sensitivity against sorafenib via measurement of spheroid size and cell viability examined by resazurin assays. The size of HCC spheroids dramatically decreased after treatment with sorafenib, whereas MCTS models grown with stromal and HCC cells displayed greater resistance to sorafenib, depending on the ratio of stromal cells in the MCTS models [Fig. 2A]. Consistent with the spheroid size, cell viability after sorafenib treatment was also substantially elevated in proportion to the ratio of stromal cells in the MCTS models [Fig. 2B].

We also characterized the effects of anticancer drugs, such as 5-FU, cisplatin, regorafenib and gefitinib on the growth of spheroids composed of Huh7 spheroids or the MCTS model to confirm their clinical significance. The results showed that these anticancer drugs were less efficacious in the MCTS model than in the Huh7 spheroids, suggesting that reciprocal crosstalk between the HCC and the TME contributed to chemoresistance in the MCTS model [Fig. 2C].

Because crosstalk between HCC cells and their TMEs is regarded as critical for the promotion of cell migration, a characteristic of cancer cells, MCTS models with ratios of HCC cells and stromal cells were tested using a tumor spheroid–based migration assay to determine the distance from the center of the MCTS models to the leading edge of the migrating HCC cells. This assay showed that crosstalk between stromal cells and HCC cells in MCTS models is an important determinant of HCC migration ability [Fig. 2D].

### Identification of compounds for HCC treatment using MCTS-based phenomic screening

**Figure 3:**
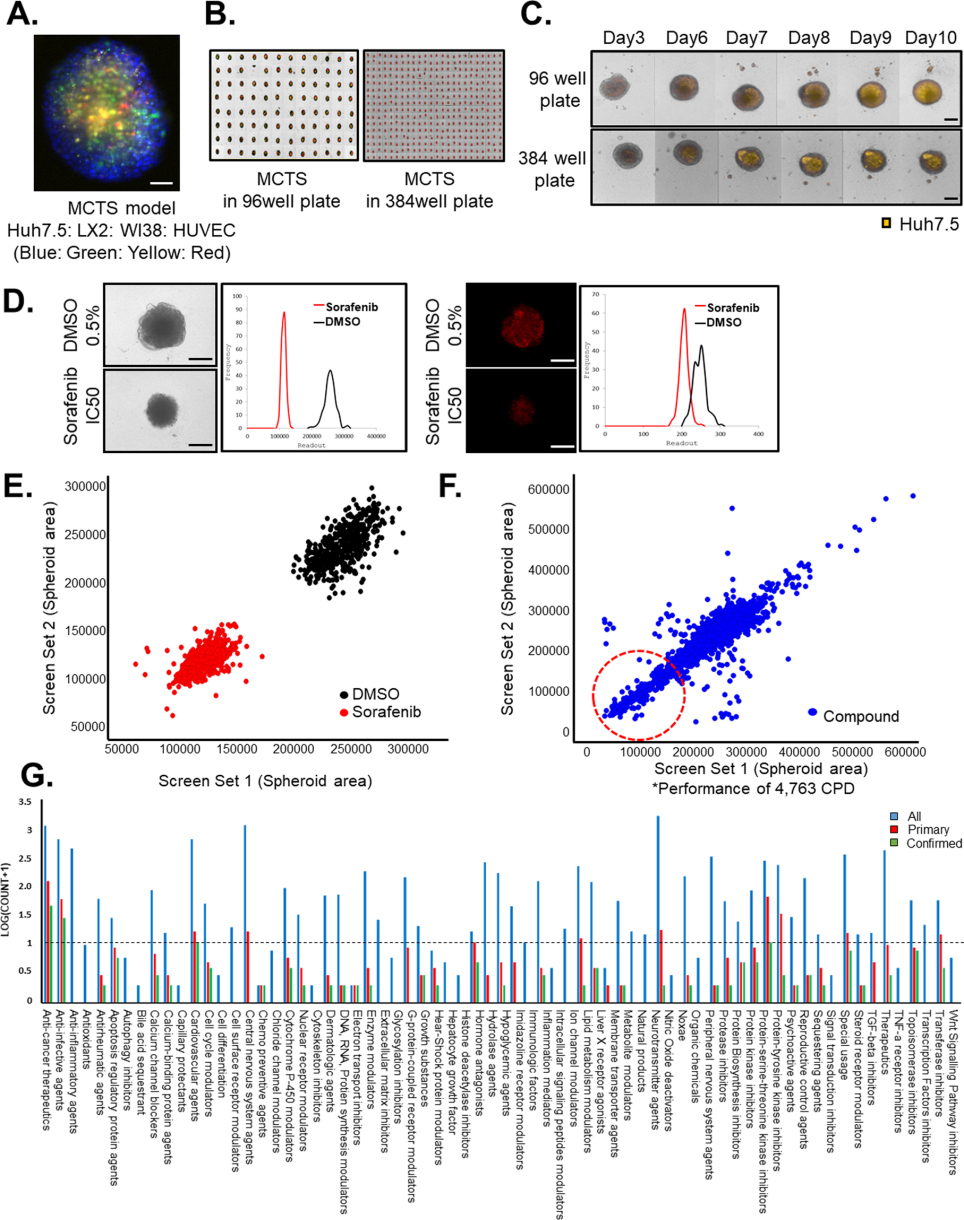
Development of a multicellular tumor spheroid (MCTS) model-based phenomic screening for hepatocellular carcinoma (HCC) drug discovery. (**A**) Homogenously configured MCTS models with Huh7.5 cells, LX2 cells, WI38 cells, and HUVECs. Scale bar: 100 µm. (**B**) Homogenous spheroids in 96- and 384-well plates. (**C**) Red fluorescent protein (RFP) intensity of Huh7.5 spheroids in 96- and 384-well plates at 10 days. Scale bar: 200 µm. (**D**) Validation of phenotypic screening using a MCTS model-based assay. Bright-field and RFP-expressing images of spheroids with sorafenib IC_50_ as a low control and 0.5% DMSO (v/v) as a high control at 10 days. Scale bar: 200 µm. (**E**,**F**) Pilot screen results. (**E**) The scatterplot analyses show positive (red; sorafenib IC_50_) and negative (black; 0.5% DMSO) controls among the validation plates. Dots represent each single well tested. (**F**) A total 4,763 compounds were screened at a concentration of 10 µM validation plates. Dots represent single tested wells. Dots are hits from the sorafenib control. The correlation coefficient (r^2^ = 0.8) was calculated by using the obtained optimal number of components. (**G**) Pharmacological actions of 4,763 compounds comprised of FDA-approved drugs and each primary and confirmed hit compounds from library. All images were obtained using the high-content screening system.

We established the most appropriate MCTS model for anticancer drug screening to mimic the HCC TME according to the analyses of HCC tissue composition [Figs. 1 and 2]. Because Huh7.5 cells were fluorescently labeled, it was possible to observe the homogenously configured MCTS and the specific effects on HCC during the screening process [Fig. 3A].

Next, we focused on the development of an MCTS-based screening platform for HCC drug discovery. To establish a MCTS-based phenomic screening platform for high-throughput/high-content screening, we developed an efficient method for generating the spheroids. We successfully generated homogenously sized and configured single spheroids in 96-well or 384-well plates. The spheroid images were acquired using the high-throughput screening system within 30 minutes. Hence, we established a simple method for establishing highly reproducible (<10% size variation) spheroids involving high speed image acquisition [Fig. 3B]. To mimic the *in vivo* growth of cancer cells, we also characterized the growth of cancer cells using multi-well plates by estimating the intensity of the RFP signals in the Huh7.5 spheroids. According to intensity of the RFP signals, the HCC population continuously increased as the MCTS cells grew in the multi-well plates [Fig. 3C]. We also developed appropriate image analysis software to detect the spheroid size and the intensity of the Huh7 cells to determine the cytotoxic effects on HCC [Fig. 3D].

We then performed a screening to identify compounds that specifically altered the intensity of the Huh7.5 cell signals in the MCTS model and determine the sizes of MCTSs. Compounds were screened at an initial concentration of 10 µM to identify decreased intensities of the RFP signal or determine the sizes of the MCTSs. Positive and negative controls were 12.5 µM sorafenib and 0.5% DMSO, respectively [Fig. 3E]. We screened all compounds in duplicate to confirm the reproducibility of the observed effects. A Z’ score of 0.46 and a correlation coefficient of 0.89 for replicate screens indicated that the assay was reliable. Eighty-seven compounds significantly inhibited the size of the MCTSs and the intensity of the RFP signals [Fig. 3F].

A collection of 4,763 compounds comprising FDA-approved drugs with known molecular targets was assembled to identify novel drugs for HCC therapy. The pharmacological actions of each compound were identified from the ChemIDPlus database (https://chem.nlm.nih.gov/chemidplus/), MESH database (https://meshb.nlm.nih.gov/search), and the SciFinder, as well as from information provided by the vendors. All pharmacological actions were accounted for and were not limited to the primary ones, as it is difficult to define primary pharmacological actions of known drugs. Primary hits involved 49 categories and confirmed hits involved 39 categories. Confirmed hits are majorly involved in categories of anticancer therapies, anti-infective agents, cardiovascular agents, and tyrosine kinase inhibitors [Fig. 3G].

### Inhibitors of Na^+^/K^+^-ATPase suppress cell growth in liver cancer

**Figure 4:**
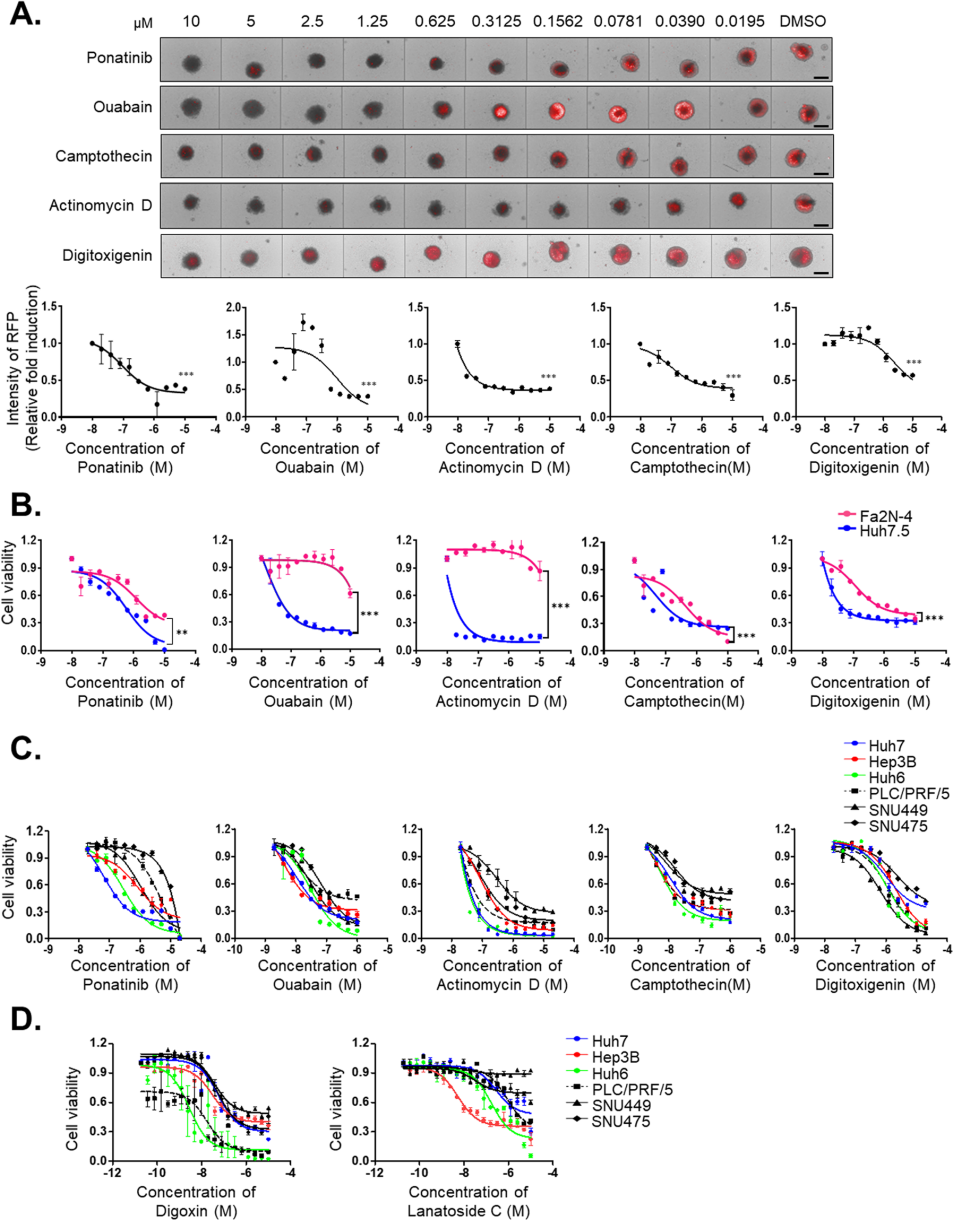
Drug sensitivities of hits from the primary screening of the multicellular tumor spheroid (MCTS), normal hepatocyte, and hepatocellular carcinoma (HCC) cell lines according to concentration. (**A**) Spheroid size and red fluorescent protein (RFP) signal intensity of the MCTS model after treatment with ponatinib, ouabain, camptothecin, actinomycin D, or digitoxigenin at the indicated concentrations (upper panel). Dose-response curve of the RFP signal intensity of the MCTS model after treatment with ponatinib, ouabain, camptothecin, actinomycin D, or digitoxigenin (lower panel). (**B**) Toxicity of the five hit compounds on normal hepatocytes (Fa2N-4) and Huh7.5 cells at the indicated concentrations in monolayer culture conditions after 48 hours of treatment. (**C**) Dose-response curve of the cell viability of 6 HCC cell lines in monolayer culture conditions after treatment with ponatinib, ouabain, camptothecin, actinomycin D, or digitoxigenin for 48 hours. (**D**) Dose-response curve of the cell viability of 6 HCC cell lines in monolayer culture conditions after treatment with digoxin, lanatoside, and lanatoside C for 48 hours. All images were obtained using the high-content screening system. Data are shown as the means and standard deviation (SD) of triplicate experiments. Scale bar: 200 µm. **p < 0.005, ***p < *0.0005*.

To verify hits from the primary screening, dose-response studies were performed to quantify the potency of selected hits using the MCTS model. Five compounds (ponatinib (IC_50_ = 92.3 nM), ouabain (IC_50_ = 0.992 μM), camptothecin (IC_50_ = 96.8 nM), actinomycin D (IC_50_ = 0.563 pM), and digitoxigenin (IC_50_ = 2.3 µM)) sufficiently inhibited the RFP signal by greater than 60% and decreased the MCTS size at concentrations below 1 µM relative to the effects of 12.5 µM sorafenib [Fig. 4A]. To avoid hepatotoxicity, dose-response curves of selected hits were also used to determine the effective concentrations needed to decrease proliferation by 50% (EC_50_) in Huh7.5 cells and in the normal Fa2N-4 hepatocyte cells. Among five compounds, ouabain, actinomycin D, and digitoxigenin had prominent anticancer efficacy without hepatotoxicity [Fig. 4B, Supplementary Table I]. To ascertain the effects of five hit compounds on human HCC cells, we measured the IC_50_ values of hit compounds on the growth of various HCC cell lines. Dose-response curves showed that the hit compounds sufficiently induced cell death in HCC cells [Fig. 4C; Supplementary Table II].

Notably, inhibitors of Na^+^/K^+^-ATPase (ouabain and digitoxigenin) act effectively as anticancer drugs in a variety of HCC cell lines. Thus, we also confirmed the anticancer efficacy of other cardiac glycosides that have a structure similar to ouabain and digitoxigenin, such as digoxin and lanatoside C. As expected, digoxin and lanatoside C also inhibited HCC growth in a concentration-dependent manner [Fig. 4D; Supplementary Table II]. Among four cardiac glycosides, we selected the most effective two drugs—ouabain and digoxin—to use for further experiments.

### Inhibitors of Na^+^/K^+^-ATPase accelerate apoptosis and attenuate migration of HCC

**Figure 5:**
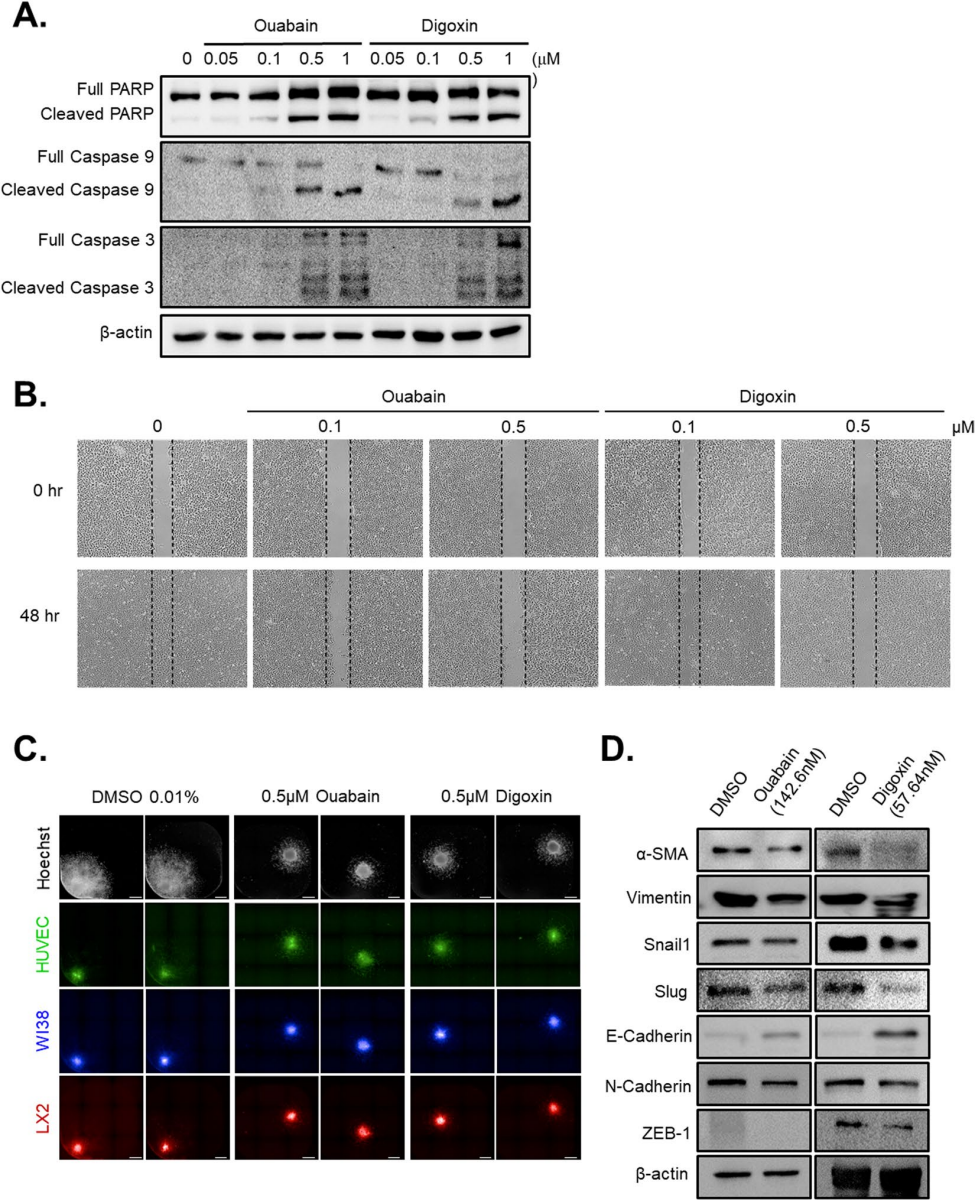
Apoptosis and migration of hepatocellular carcinoma (HCC) and multicellular tumor spheroid (MCTS) cells induced by inhibitors of Na^+^/K^+^-ATPase. (**A**) Expression of apoptosis-related protein (cleaved poly [ADP-ribose] polymerase, cleaved caspase-9, cleaved caspase-3) in ouabain and digoxin-treated MCTS at indicated concentration. (**B**) Migration capacity of Huh7 cells when they were treated with ouabain and digoxin at indicated concentration for 48 hr. (**C**) Migration capacity of HCC MCTS (green: HUVEC; blue: WI38; red: LX2; white: all stromal cells and cancer) when they were treated with 0.5 uM of ouabain and digoxin for 96 hr. Scale bar: 100 µm. (**D**) Expression levels of epithelial-to-mesenchymal–related protein in ouabain (left panel) and digoxin (right panel) treated MCTS at indicated concentration. All images were obtained using the high-content screening system. Data are shown as the means and standard deviation (SD) of triplicate experiments.

Ouabain and digoxin sufficiently upregulated apoptosis markers, such as the cleavage of poly (ADP-ribose) polymerase, caspase-3, and caspase-9, from a concentration of 0.1 μM in HCC-MCTS [Fig. 5A, Supplementary Fig. 6]. Moreover, we estimated the metastatic capacity of HCC after treatment with ouabain and digoxin. The wound healing assay revealed that 0.1 μM ouabain and digoxin can inhibit the metastatic capacity of HCC [Fig. 5B, Supplementary Fig. 7].

In Fig. 2D, we showed that crosstalk between stromal cells and HCC cells in MCTS models was important for the regulation of HCC migration ability. The MCTS-based migration assay revealed that increased migration capacity of HCC from crosstalk between stromal cells and HCC cells in MCTS was also significantly attenuated by treatment with ouabain and digoxin [Fig. 5C]. E-cadherin and N-cadherin can mediate cell-cell adhesion and modulate cell migration and tumor invasiveness. Both drugs displayed an upregulation of E-cadherin expression and a loss of N-cadherin expression and inhibited the expression of EMT-related molecules such as α-SMA, Vimentin, Snail, Slug and ZEB-1 [Fig. 5D].

These results indicated that inhibitors of Na^+^/K^+^-ATPase can induce HCC cell death and migration capacity from crosstalk between stromal cells and HCC cells in MCTSs.

### The MCTS-derived xenograft mouse model can reappear *in vivo* HCC

**Figure 6:**
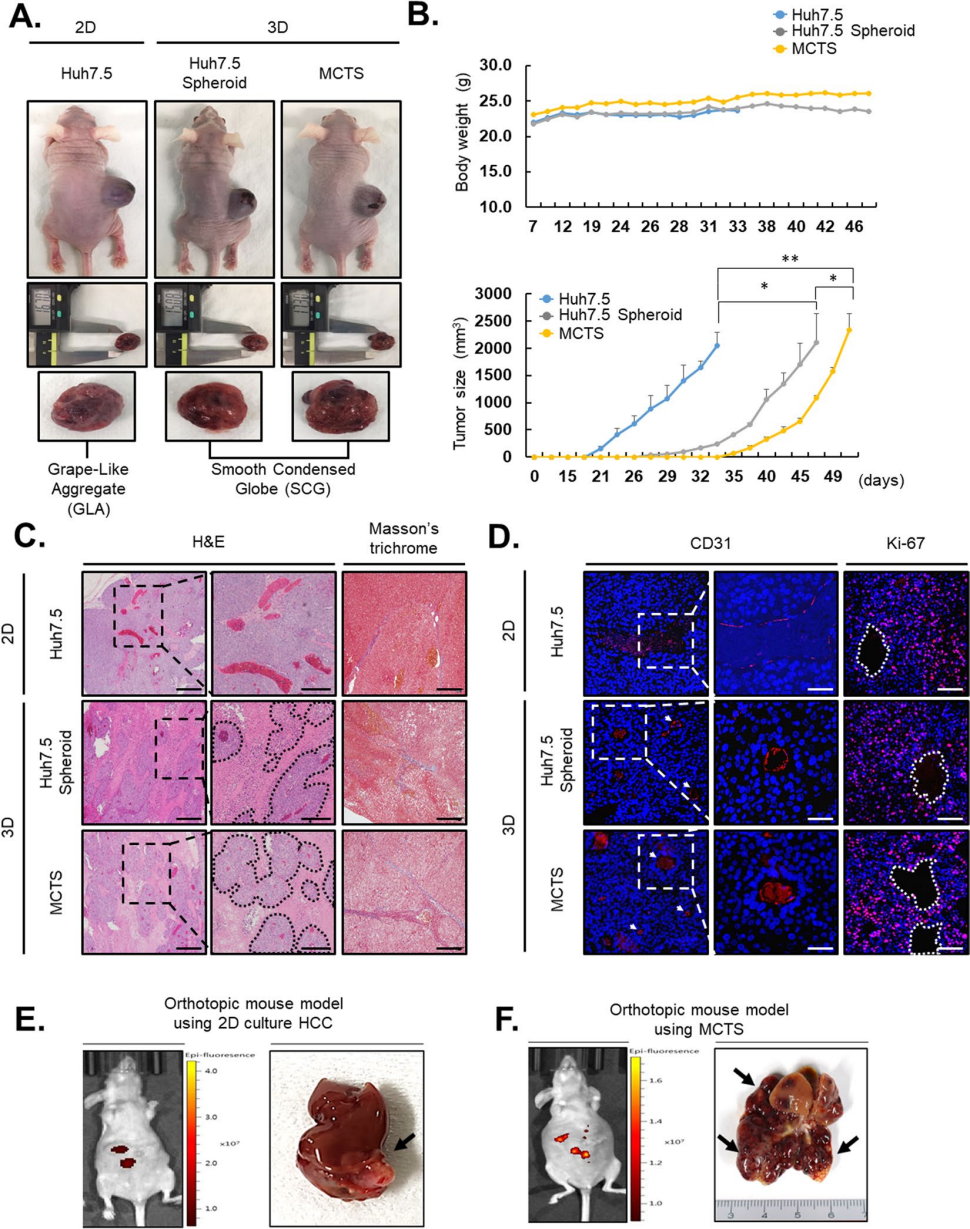
Multicellular tumor spheroid (MCTS)-derived xenograft model as a novel and effective tool for the growth of human hepatocellular carcinomas in mouse models. (**A**) MCTS-derived xenograft model established by injecting Huh7.5 cells, Huh7.5 spheroids, or MCTSs subcutaneously into nude mice. (**B**) Animal body weight and tumor volume were measured twice per week until a tumor size of 2,000 mm^3^ was reached. *p < 0.5, **p < *0.05* (**C**) Representative images of hematoxylin and eosin- or Masson’s trichrome-stained tumors from Huh7.5, Huh7.5 spheroids, or MCTS-derived xenografts. The inset shows macroscopic images of the corresponding tumor samples. Scale bar: 100 µm. (**D**) Immunofluorescence staining of CD31 and Ki-67 expression and nuclei in tumor sections of Huh7.5, Huh7.5 spheroids, or MCTS model-derived xenografts. The inset shows detailed areas containing blood vessels. (**E**,**F**) Orthotopic mouse model of Huh7.5 (**E**) and Huh7.5-MCTS (**F**). Intensity of fluorescence using liver animal imaging system (IVIS spectrum) (left panel) and liver tissue (right panel). The arrow indicates the tumor. Scale bar: 100 µm.

To show that MCTS models mimicked the TME, we made subcutaneous xenografts by monolayer-cultured Huh7.5 cells, Huh7.5 spheroids, or MCTS model systems in nude mice. General xenograft models, which were implanted harvested cells from monolayer cultures, formed relatively white and grape-like aggregated tumors. In contrast, spheroid-derived xenograft models formed deep red and smoothly condensed, globe type tumors [Fig. 6A]. When we performed comparison analyses of tumor growth among Huh7.5 cells, Huh7.5 spheroids, and MCTS models, all tumor volumes in the xenograft models were increased in the following order without changes in animal body weight [Fig. 6B, upper panel]. Although the formation of seed tumors was faster in Huh7.5 cells than in Huh7.5 spheroids and the MCTS models, tumor growth was faster in the spheroid culture system after formation of seed tumors [Fig. 6B, lower panel].

We dissected three types of tumor tissues from monolayer-cultured Huh7.5 cells, Huh7.5 spheroids, or MCTS-xenograft mice, then performed H&E staining. The staining revealed that each of the three kinds of tissues had different forms, structures, and vascular distributions [Fig. 6C, left]. In Masson’s trichrome staining, a difference in fibrotic degree was found among the three types of tumor tissues from monolayer-cultured Huh7.5 cells, Huh7.5 spheroids, or MCTS-xenograft mice. Late-stage fibrosis was observed in tissues from the MCTS-xenograft mice [Fig. 6C, right]. Antibody to CD31 was primarily used to confirm the presence of vascular endothelial cells in histological tissue sections. CD31 was distributed irregularly over a relatively large area in tumor tissues from monolayer-cultured Huh7.5-xenograft mice, whereas tumor tissues from Huh7.5 spheroids or MCTS-xenograft mice showed that CD31 is expressed in a compartmentalized form [Fig. 6D, left].

To measure tumor cell proliferation around blood vessels, we also performed immunohistochemical analyses of Ki67 in tumor tissues. Notably, tumor tissues from MCTS-xenograft mice showed the highest expression of Ki67 around blood vessels among the three tissues [Fig. 6D, right].

Next, monolayer-cultured Huh7 cells and MCTSs, which included same number of HCC cells, were orthotopically transplanted into mice for characterization of MCTS models [Fig. 6E,F]. Orthotopic mouse models using MCTS were seriously affected by liver ascites, with scarified livers so damaged that it was difficult to recognize the shape, and were covered by a large amount of cancer cells relative to the orthotopic mouse model using 2D culture HCC. Taken together, the results showed that the shape and configuration of tumor tissues are vary by the method of cell culture for transplantation. In particular, the MCTS-derived xenograft model was shown to be very similar to *in vivo* HCC.

### Inhibitors of Na^+^/K^+^-ATPase suppress cell growth in liver cancer *in vivo*

**Figure 7:**
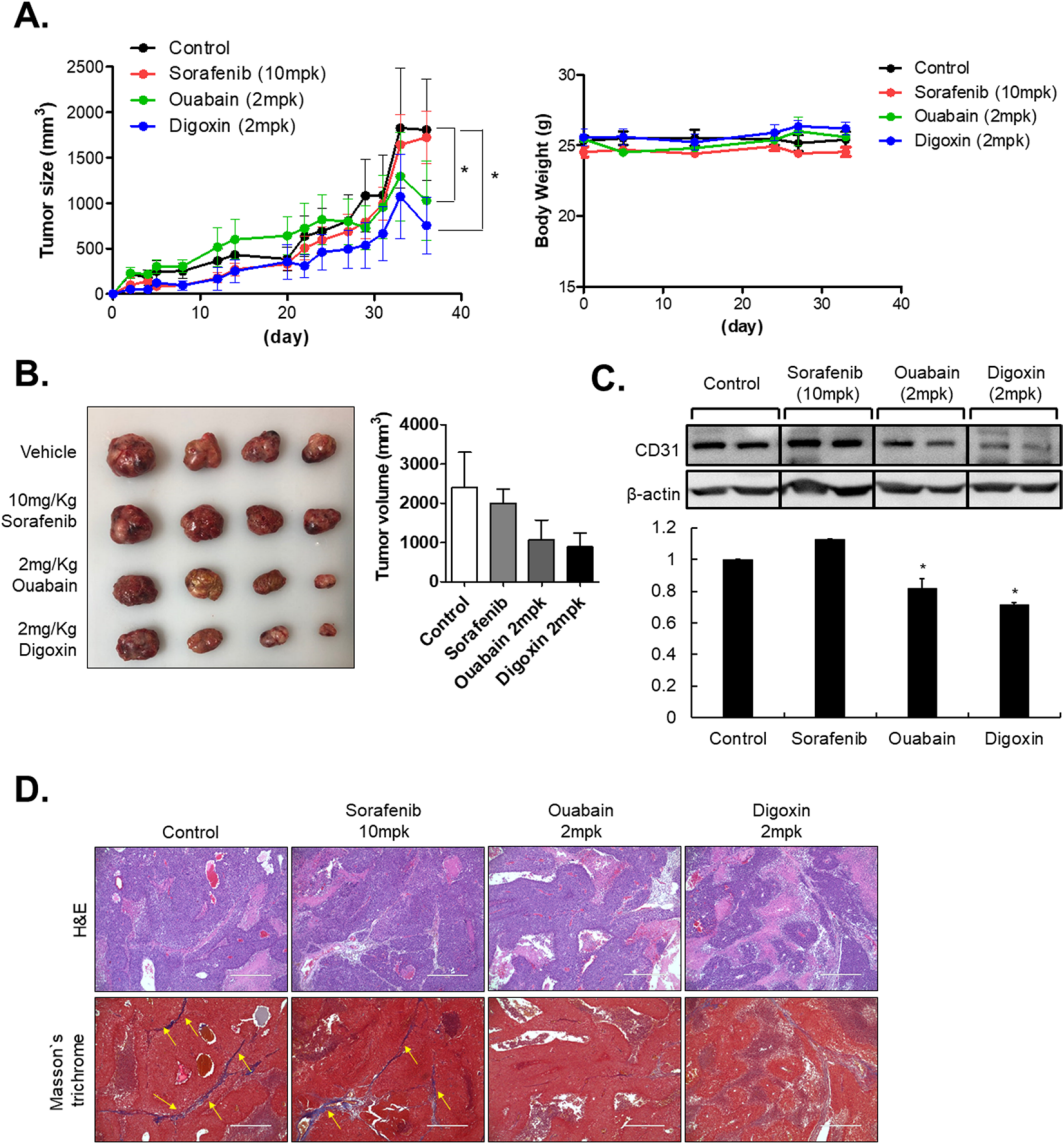
Treatment with inhibitors of Na^+^/K^+^-ATPase suppress tumor growth HCC- Multicellular tumor spheroid (MCTS) xenograft *in vivo* (**A**) MCTS-derived xenograft mice tumor volume and body weight. (**B**) Representative image of tumors resected from MCTS-derived xenograft mice. (**C**) Expression of CD31 in sorafenib, ouabain and digoxin injected xenograft mice. (**D**) Representative images of hematoxylin and eosin- or Masson’s trichrome-stained tumors after treatment of inhibitors of Na^+^/K^+^-ATPase and sorafenib. Scale bar: 200 µm.

Next, we transplanted HCC-MCTSs into BALB/c nude mice to analyze the capability of ouabain and digoxin to suppress HCC. Ouabain and digoxin were treated by Oral administration and sorafenib was injected by intraperitoneal (i.p). As the results, treatments of ouabain and digoxin significantly reduced tumor volume without loss of body weight relative to sorafenib-treated mice [Fig. 7A,B]. Generally, ouabain and digoxin dosages used in the current *in vivo* experiments did not cause cardiac toxicity^20,21^.

Because certain inhibitors of Na^+^/K^+^-ATPase could attenuate angiogenesis^22,23^, we also confirmed the anti-angiogenic effect of ouabain and digoxin by our *in vivo* studies. Western blot analysis of tumor tissues showed that treatment of ouabain and digoxin sufficiently inhibited expression of CD31, an angiogenesis-related marker, relative to sorafenib treatment *in vivo* [Fig. 7C].

H&E staining showed ouabain and digoxin reduced tumor burden and Masson’s trichrome staining showed that treatment of Na^+^/K^+^-ATPase inhibits more significantly attenuated the degree of fibrosis than sorafenib treatment in MCTS-xenograft mice [Fig. 7D]. Taken together, the results showed that treatment with inhibitors of Na^+^/K^+^-ATPase inhibits EMT and facilitates a robust anticancer activity against HCC.

## Discussion

HCC is the sixth most common malignancy and the second leading cause of cancer-related deaths in the world. The highest incidence occurs in Eastern Asia and Sub-Saharan Africa. In addition, liver cancer incidence and mortality rates in The Republic of Korea rank as the highest in the world^24^.

The first-line therapy for HCC is surgical resection of the tumor, and the second-line therapy is treatment with the kinase inhibitor, sorafenib. However, both therapies are only partially effective in patients with liver cancer. In addition, HCC rapidly becomes sorafenib-resistant^25–27^. To improve treatment options, additional drugs were developed via the sorafenib mode of action; however, these efforts still have not resulted in significantly better efficacies. Based on clinical considerations, we developed an MCTS model, a novel and effective approach to mimic tumor complexity and heterogeneity, to screen for compounds against HCC.

Differences in sensitivities to sorafenib between unicellular tumor spheroids and patient-derived tumors emphasized the importance of crosstalk between the TME and HCC to determine chemoresistance in patient-derived tumor spheroids [Fig. 1A–C]. We also characterized the effects of sorafenib on the growth of Huh7 spheroids or MCTSs, which were composed of HCC cells, fibroblasts, HSCs, cancer stem cells, and vascular endothelial cells. This system mimicked the HCC TME based on analyses of the HCC tissue composition and showed that sorafenib was less efficacious in MCTSs than in Huh7 spheroids [Fig. 2A]. We also confirmed the efficacies of various anticancer drugs and cytotoxic drugs using HCC spheroids and the MCTS model. As expected, the MCTSs showed a stronger resistance to cytotoxic drugs than HCC spheroids [Fig. 3C]. Taken together, these results suggested that reciprocal crosstalk between HCC and the TME in 3D culture systems is an important consideration in the development of an effective drug screening process.

Various types of MCTS models have been used to characterize the crosstalk between tumor and stromal cells. Co-cultures of HUVECs and hepatocytes in heterocellular 3D systems have been used to monitor cancer angiogenesis^16,28^. Co-culturing of prostate cancer epithelial and stromal cells in 3D conditions influenced the secretion of E-cadherin^29^. A co-culture system of 3D cloned cancer cells and stromal fibroblasts also showed a stronger invasive phenotype than 3D cloned cancer cells alone^30^. Dynamic analyses of HCC MCTS formation has shown the fundamental role of E-cadherin and β1-integrin in cell aggregation and multicellular tumor spheroid compaction^17^. Many studies have reported that the co-cultures of HCC and stromal cells enhance cancer progression via activation of specific signal pathways and alterations of cytokine expression profiles^15,31–33^.

To characterize the causes of drug resistance related to the TME, sophisticated methodologies must be developed to reflect the TME. In the present study, we showed that the MCTS model closely mimicked the *in vivo* TME. First, gene expression profiling and GSEA showed that MCTS-specific signatures were enriched in HCC tumor tissues from 60 patients [Fig. 1H]. Second, we established MCTS-derived xenograft models. Macroscopically, liver cancer appears as a nodular or infiltrative tumor^34^. The nodular tumor type may be solitary (large mass) or multiple (i.e., when developed as a complication of cirrhosis). Tumor nodules are round to oval in shape and are circumscribed. The diffuse nodular type is poorly circumscribed and infiltrates the portal or hepatic veins. MCTS-derived xenograft model is more similar to the *in vivo* nodular type of HCC than the monolayer-cultured cancer cell and Huh7.5 spheroids-derived xenograft model [Fig. 6].

However, it is not known whether the cells removed from patients with liver cancer and propagated *in vitro* have the same characteristics as those *in vivo* with respect to the tumor type, signaling, and the state of the cells. Therefore, research exploring the dynamic changes of the TME within patient tissues is essential, as is extensive collaborations with basic researchers and clinical pathologists.

In the present study, we successfully established an MCTS-based screening platform, which generated homogenously sized and configured single spheroids in multi-well plates [Fig. 3]. After the MCTS-based screening, 87 compounds were identified as hits that fulfilled the criteria of selectivity [Fig. 3G]. Using hit validations and secondary assays, we finally identified five compounds that sufficiently inhibited the RFP signal by greater than 60% and decreased the MCTS model size at a lower concentration (1 µM) than sorafenib (12.5 µM) used as a positive control [Fig. 4]. These five positive compounds were divided into two groups: anticancer drugs and cardiac glycosides. Ponatinib, camptothecin, and actinomycin D were not further considered because these drugs are known to be highly effective anticancer agents. Ponatinib, a FDA-approved tyrosine kinase inhibitor, was identified in 2012 as a candidate for the treatment of chronic myeloid leukemia and Philadelphia chromosome-positive acute lymphocytic leukemia^35,36^. Camptothecin is a cytotoxic quinoline alkaloid that inhibits topoisomerase I^37^. Camptothecin has attracted considerable clinical attention on the basis of its promising antitumor activity in many *in vitro* and *in vivo* studies^38^. Actinomycin D intercalates into DNA, preventing the activity of RNA polymerases^39,40^. Actinomycin D is the first antibiotic used to treat cancers such as gestational trophoblastic neoplasia, Wilms tumor, rhabdomyosarcoma, Ewing’s sarcoma, and acute myeloid leukemia^41^. The mechanism by which actinomycin D causes tumor cell death is not fully known.

In the present study, cardiac glycosides were of interest, especially in terms of drug repositioning. Ouabain and digoxin are cardiac glycosides, which are naturally occurring compounds sharing the ability to inhibit the plasma membrane Na^+^/K^+^-ATPase and indirectly promote the intracellular accumulation of Ca^2+^ ions^42^. In this study, ouabain and digoxin accelerate apoptosis and attenuate migration of HCC [Figs. 5 and 7]. Cancer cells are more vulnerable to the effects of cardiac glycosides than normal cells because activation of the Src-epidermal growth factor receptor-mitogen-activated protein kinase pathway by cardiac glycosides in cancer cells, unlike in normal cells, results in growth arrest through an increased expression of the cyclin-dependent kinase inhibitor 1 A (p21^CIP1^)^43^. Recently, cardiac glycoside-based anticancer drugs, such as Anvirzel and UNBS1450, have undergone testing in clinical trials^44^. Ouabain has anti-proliferative and/or apoptotic effects *in vitro* in a wide range of cancer types such as breast cancer, prostate cancer, lung cancer, melanoma, and leukemia^45–47^. In terms of HCC, ouabain induces HepG2 cell apoptosis through the generation of reactive oxygen species and cell cycle S phase arrest by decreasing the activities of cyclinA1/cyclin-dependent kinase 2 and aurora A kinase^48,49^. However, digoxin only displays anticancer effects in myeloma and renal cancer^50^. Recently, digoxin is attracting attention as a therapeutic agent for NASH via regulation of HIF-a transcriptional activity^21^. Moreover, cardiac glycosides similar in efficacy to digoxin could reduce liver injury^21^, inhibitors of Na^+^/K^+^-ATPase are expected to be liver cancer therapeutics without hepatotoxicity [Figs. 4, 5 and 7]. The mechanism by which ouabain and digoxin cause HCC cell death in MCTS models is not yet known. Functional studies of ouabain and digoxin will hopefully provide new targets that can be used for the development of novel anticancer drugs in patients with liver cancer.

In the present study, because liver cancer contains abundant fibrosis, we developed a MCTS model system with a mixture of HCCs, HSCs, fibroblasts, and vascular endothelial cells to mimic the *in vivo* microenvironment of liver cancer. Although this MCTS model is still preliminary and does not fully reflect the *in vivo* microenvironment because of the absence of an immune response, it is a highly useful method to elucidate the roles of the TME on tumorigenesis and liver fibrosis. Furthermore, the 3D TME of the MCTS models will provide a new paradigm for high-throughput drug screening that will significantly improve the efficiency of identifying new drugs for liver cancer treatment. The MCTS-based phenomic screening presented inhibitors of Na^+^/K^+^-ATPase as a novel target to sensitize HCC cells.

### Ethics approval and consent to participate

The animal protocol was approved by the institutional animal care and use committee of ASAN medical center and conducted strictly in accordance to the national institute of health guide for the care and use of laboratory animals.

### Consent for publication

All authors read and approved the final manuscript for publication.

## Supplementary information

Supplementary Figure and legend.
