# Supplementary material for: Inhibitors of Na+/K+ ATPase exhibit antitumor effects on multicellular tumor spheroids of hepatocellular carcinoma
Source: Sci Rep. 2020 Mar 24;10:5318. doi: 10.1038/s41598-020-62134-4 (PMC7093469; doi:10.1038/s41598-020-62134-4)

Inhibitors of Na^+/K^+ ATPase exhibit antitumor effects on multicellular tumor spheroids of hepatocellular carcinoma

Yeonhwa Song[1], Su-Yeon Lee[1], Sanghwa Kim[1], Inhee Choi[2], Se-Hyuk Kim[1], David Shum[3], Jinyeong Heo[3], A-Ram Kim[1], Kang Mo Kim[4] and Haeng Ran Seo[1*]

[1] Cancer Biology Laboratory, [2] Medicinal Chemistry, [3] Screening Discovery Platform, Institut Pasteur Korea, 16, Daewangpangyo-ro 712 beon-gil, Bundang-gu, Seongnam-si, Gyeonggi-do, 13488, Korea, [4]Department of Gastroenterology, Asan Liver Center, Asan Medical Center, University of Ulsan College of Medicine, Olympic-ro 43-gil 88, Songpa-gu, Seoul, 05505, Korea

*Corresponding author: Haeng Ran Seo, Cancer Biology Research Laboratory, Institut Pasteur Korea, 16, Daewangpangyo-ro 712 beon-gil, Bundang-gu, Seongnam-si, Gyeonggi-do, 13488, Republic of Korea; Tel: +82-31-8018-8300; Fax: +82-31-8018-8031; E-mail: shr1261@ip-korea.org

Supplementary Table I. IC_{50} value of HIT compounds on the growth of Fa2N-4 and Huh7.5 cell lines

	**Fa2N-4**	**Huh7.5**
**Ponatinib**	1.2 µM	0.56 µM
**Ouabain**	13.5mM	12.75 µM
**Camptothecin**	0.44 µM	47.31nM
**Actinomycin D**	2.63mM	0.0013nM
**Digitoxigenin**	0.11 µM	0.024nM

Supplementary Table II. IC_{50} value of HIT compounds on the growth of various HCC cell lines.

	**Ponatinib**	**Ouabain**	**Camptothecin**	**Actinomycin D**	**Digitoxigenin**	**Digoxin**	**Lanatoside C**
**Huh7**	64.95 nM	10.96 nM	10.96 nM	0.997 nM	0.98 µM	57.64nM	277.7nM
**Hep3B**	0.83 µM	4.02 nM	4.02 nM	85.87 nM	2.04 µM	30.11nM	5.373nM
**Huh6**	0.29 µM	30.61 nM	4.04 nM	1.29 pM	1.03 µM	2.742nM	174.1nM
**PLC/PRF/5**	5.3 µM	14.17 nM	3.35 nM	6.33 nM	1.24 µM	17.74nM	850.9nM
**SNU449**	1.1 µM	29.48 nM	8.24 nM	295.5 nM	0.67 µM	38.02nM	> 10 µM
**SNU475**	5.32 µM	66.69 nM	15.9 nM	86.05 nM	2.34 µM	53.44nM	> 10 µM

Supplementary Figure Legends

Supplementary Figure 1. Immunohistochemically analysis of CD44 and FAP of consecutive sections of the normal and liver cancer tissues.

Supplementary Figure 2. Biological processes significantly overrepresented in the lysates of Huh7.5 spheroids (blue) compared to multicellular tumor spheroids (MCTSs) (orange) represented in the stacked bar charts.

Supplementary Figure 3. Enrichment and depletion of molecular functions in the lysates of Huh7.5 spheroids compared to multicellular tumor spheroids (MCTSs).

Supplementary Figure 4. Enrichment and depletion of biological processes in the lysates of Huh7.5 spheroids compared to multicellular tumor spheroids (MCTSs).

Supplementary Figure 5. Enrichment and depletion of biological pathways in the lysates of Huh7.5 spheroids compared to multicellular tumor spheroids (MCTSs).

Supplementary Figure 6. Apoptosis of multicellular tumor spheroid (MCTS) induced by inhibitors of NA^+/K^+-ATPase.

Supplementary Figure 7. Migration capacity of Hep3B cells and SNU449 cells when they were treated with ouabain and digoxin at indicated concentration for 48 hr.

Supplementary Figure 1

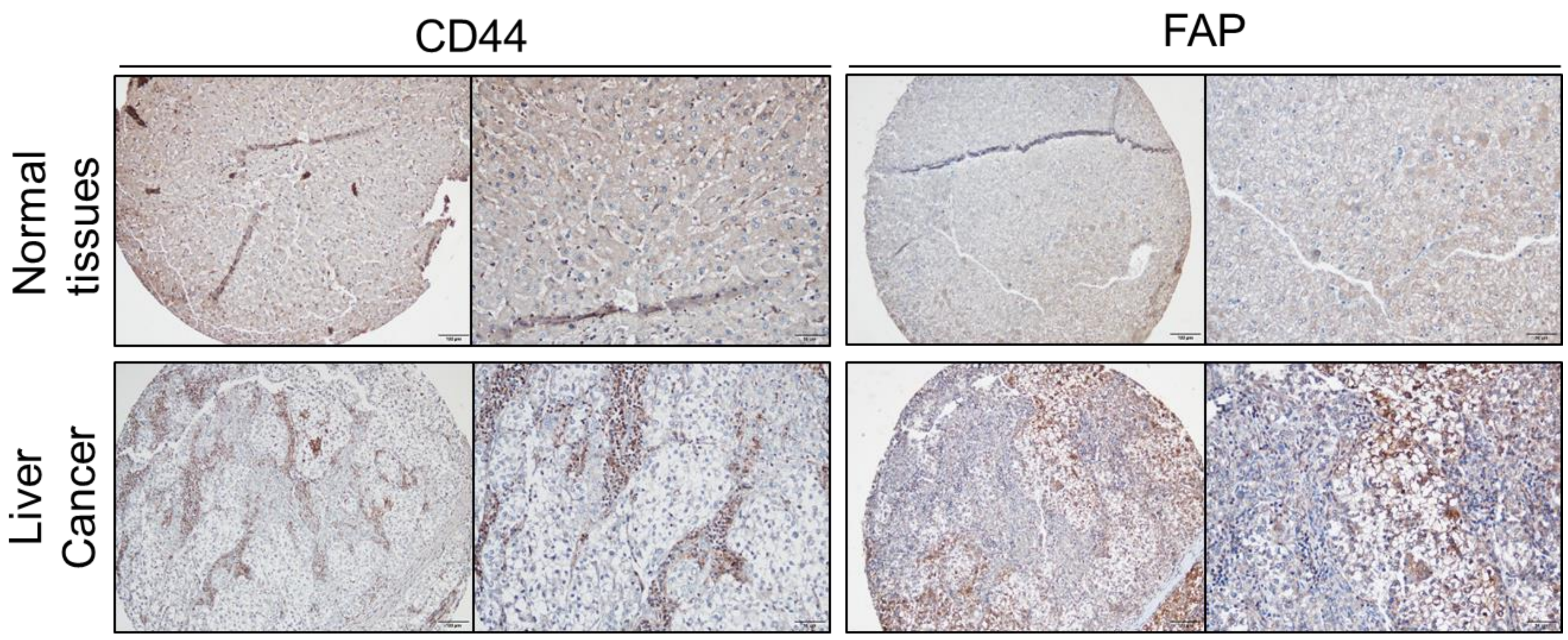

Supplementary Figure 2

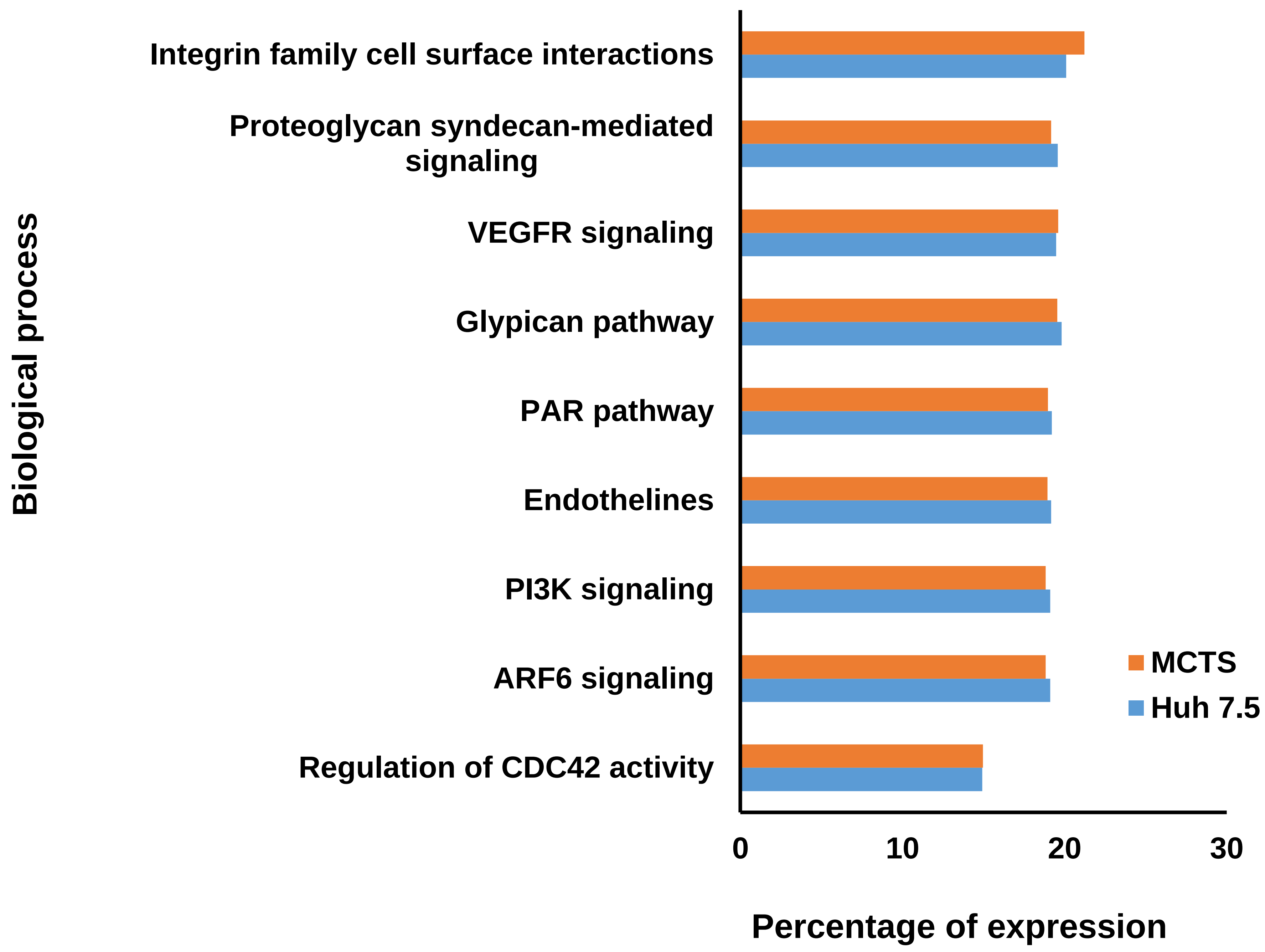

Supplementary Figure 3

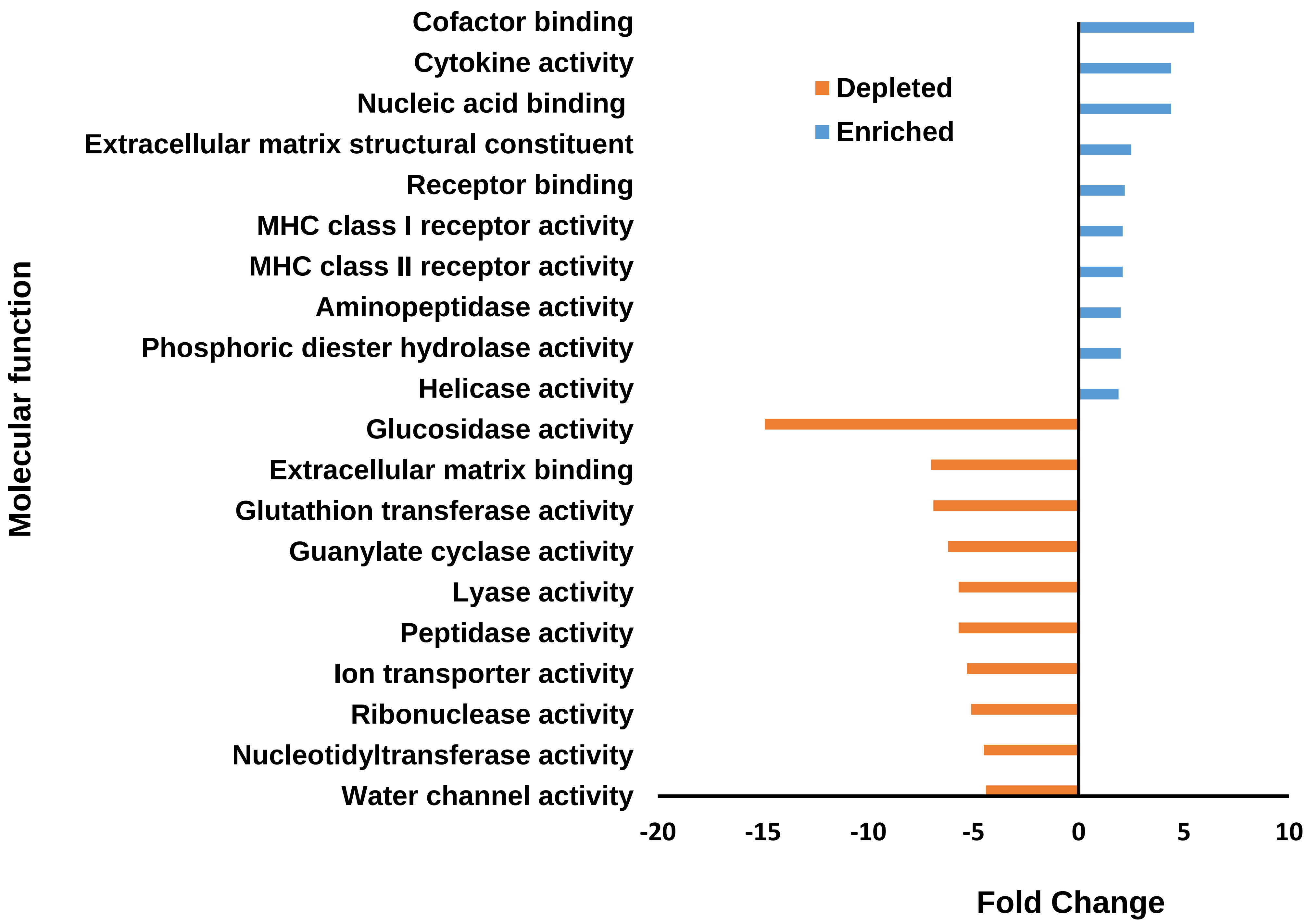

Supplementary Figure 4

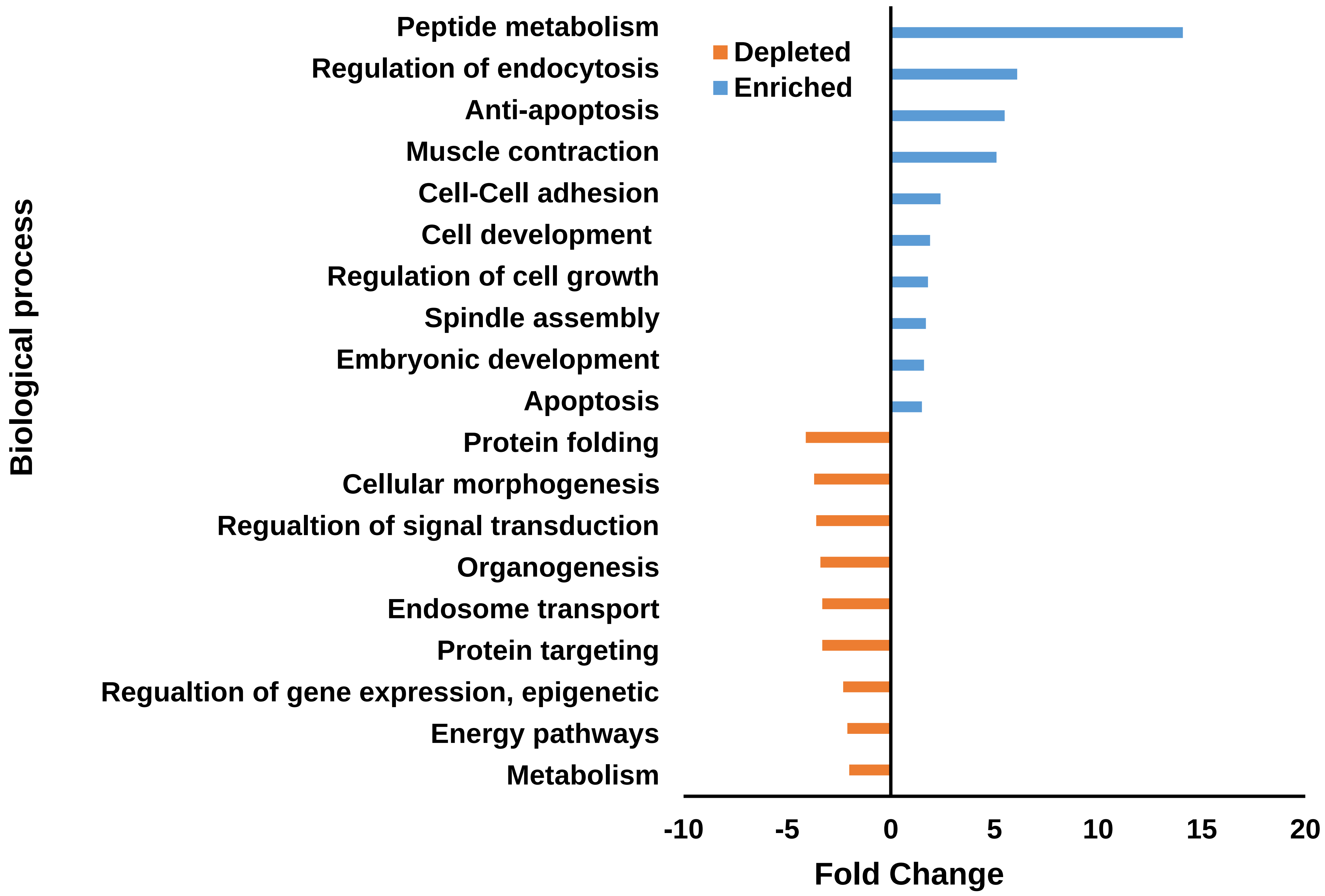

Supplementary Figure 5

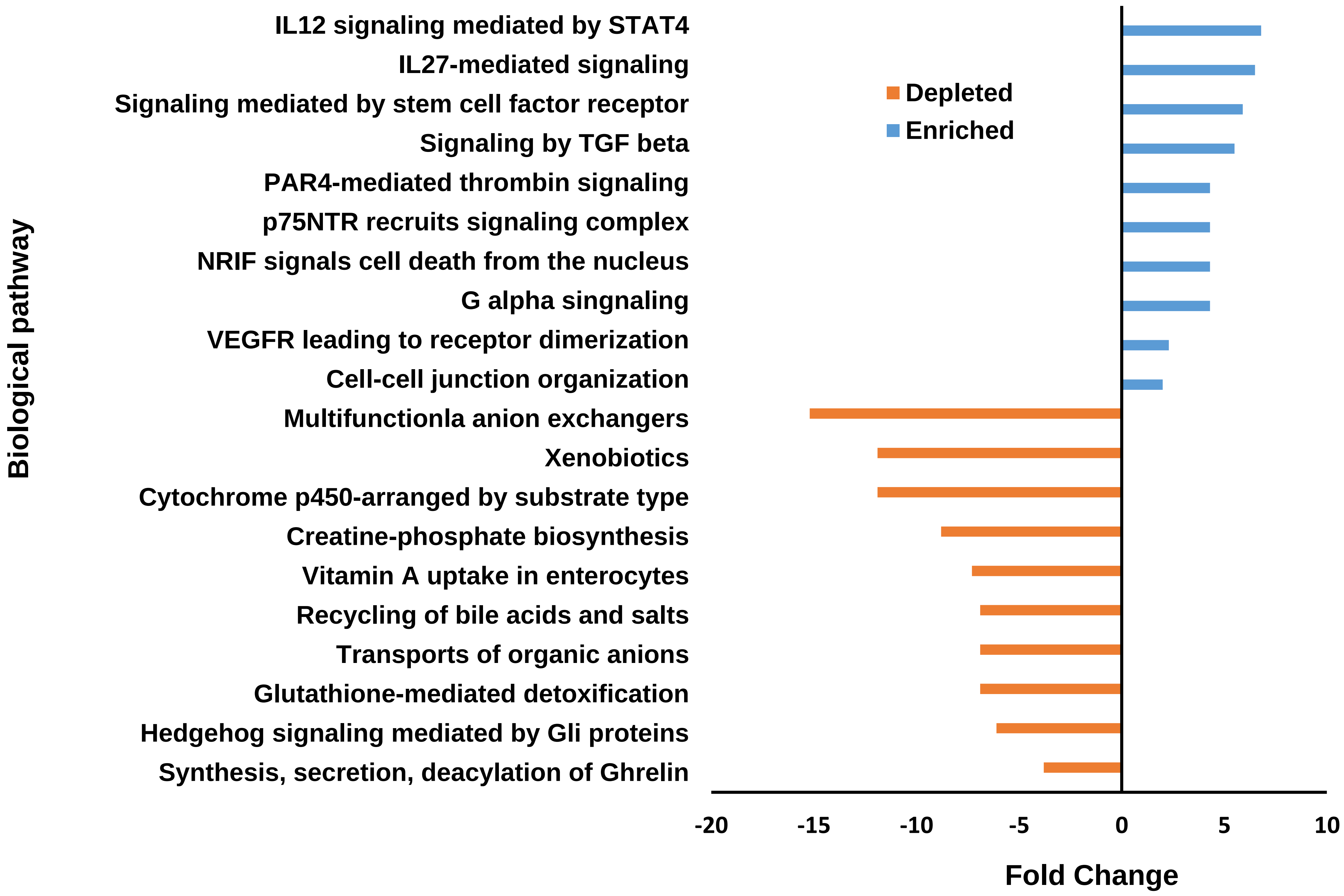

Supplementary Figure 6

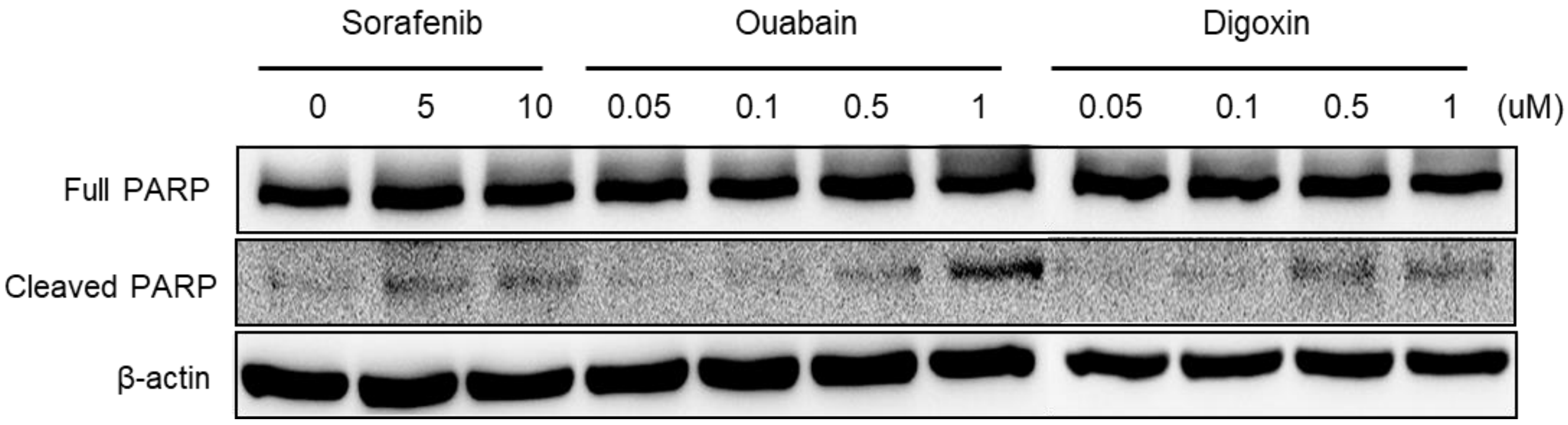

Supplementary Figure 7

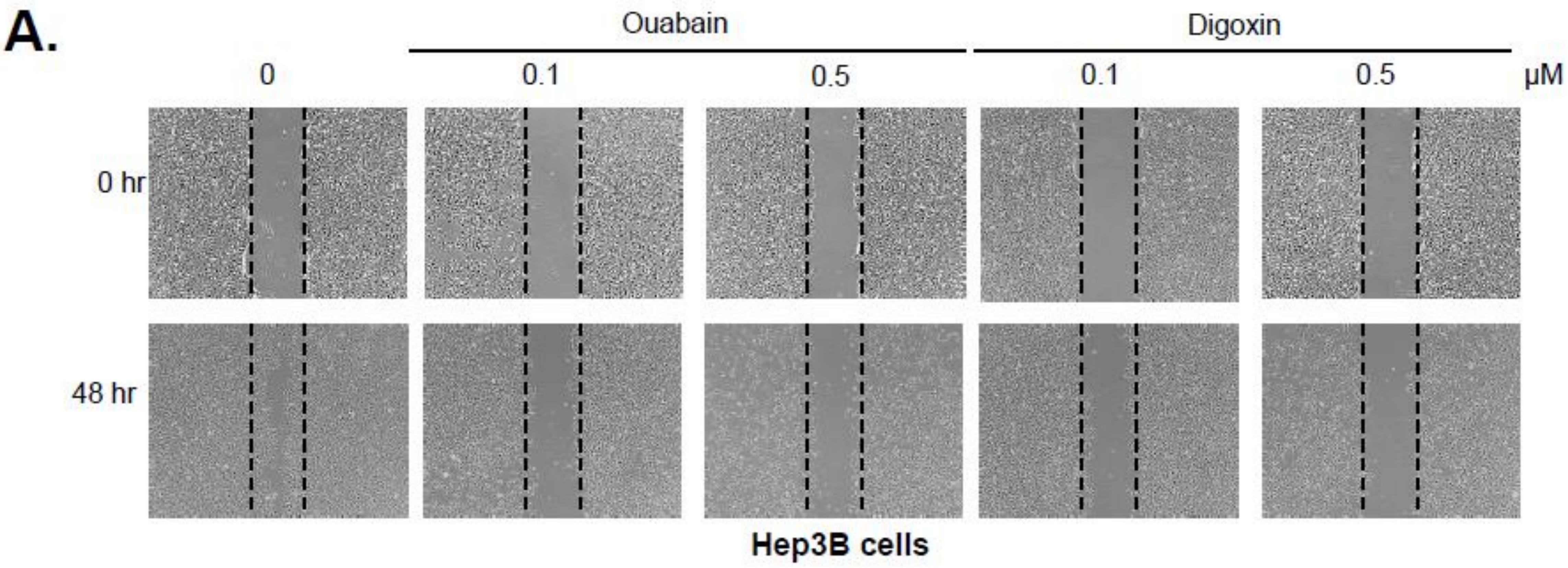

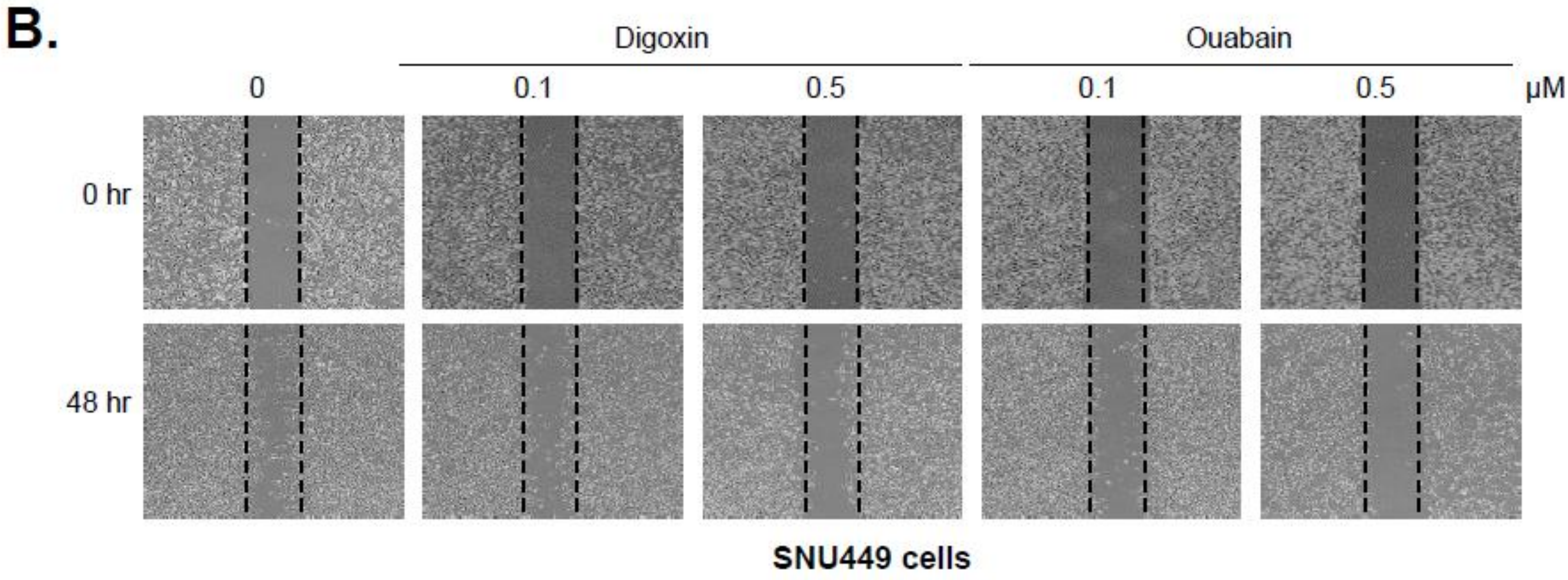

Supplement: Supplementary file 1 — Supplementary Figure and legend. [file 41598_2020_62134_MOESM1_ESM.pdf]
